# Isolated Soy Protein Supplementation and Exercise Improve Fatigue-Related Biomarker Levels and Bone Strength in Ovariectomized Mice

**DOI:** 10.3390/nu10111792

**Published:** 2018-11-17

**Authors:** Che-Li Lin, Mon-Chien Lee, Yi-Ju Hsu, Wen-Ching Huang, Chi-Chang Huang, Shih-Wei Huang

**Affiliations:** 1 Graduate Institute of Sports Science, National Taiwan Sport University, Taoyuan 33301, Taiwan; 11010@s.tmu.edu.tw (C.-L.L.); 1061304@ntsu.edu.tw (M.-C.L.); 1041302@ntsu.edu.tw(Y.-J.H.); 2 Department of Orthopedic Surgery, Shuang Ho Hospital, Taipei Medical University, New Taipei City 23561, Taiwan; 3 Department of Exercise and Health Science, National Taipei University of Nursing and Health Sciences, Taipei 11219, Taiwan; wenching@ntunhs.edu.tw; 4 Department of Physical Medicine and Rehabilitation, Shuang Ho Hospital, Taipei Medical University, Taipei 23561, Taiwan; 5 Department of Physical Medicine and Rehabilitation, School of Medicine, College of Medicine, Taipei Medical University, Taipei 11031, Taiwan

**Keywords:** branched-chain amino acid, isolated soy protein, osteoporosis, exercise training, sarcopenia

## Abstract

Isolated soy protein (ISP) is a well-known supplement and has been reported to improve health, exercise performance, body composition, and energy utilization. ISP exhibits multifunctional bioactivities and also contains branched-chain amino acids (BCAAs), which have been confirmed to positively affect body weight (BW) regulation and muscle protein synthesis. The combined effects of BCAA supplements and exercise in older postmenopausal women with osteoporosis, sarcopenia, and obesity have been inadequately investigated. Therefore, in this study, we evaluated the potential beneficial effects of soy protein supplementation and exercise training on postmenopausal mice. Forty mice (14 weeks old) with ovariectomy-induced osteosarcopenic obesity were divided into five groups (*n* = 8), namely sham ovariectomy (OVX, control), OVX, OVX with ISP supplementation (OVX+ISP), OVX with exercise training (ET, OVX+ET), and OVX with ISP and ET (OVX+ISP+ET). The mice received a vehicle or soy protein (3.8 g/kg BW) by oral gavage for four weeks, and the exercise performance (forelimb grip strength and exhaustive swimming time) was evaluated. In the biochemical profiles, we evaluated the serum glucose level and tissue damage markers, such as lactate, ammonia, glucose, blood urine nitrogen (BUN), and creatinine phosphate kinase (CPK). The body composition was determined by evaluating bone stiffness and muscle mass. All data were analyzed using one-way repeated measures analysis of variance. The physical performance of the OVX+ISP+ET group did not differ from that of the other groups. The OVX+ISP+ET group exhibited lower levels of serum lactate, ammonia, CPK, and BUN as well as economized glucose metabolism after an acute exercise challenge. The OVX+ISP+ET group also exhibited higher muscle mass and bone strength than the OVX group. Our study demonstrated that a combination of ISP supplementation and exercise reduced fatigue and improved bone function in OVX mice.

## 1. Introduction

In women, aging causes menopause-related hormonal changes that result in body fat redistribution and loss of muscle mass and strength [1]. Age-related muscle mass loss and decreased strength are termed sarcopenia, which is caused by multiple contributing factors such as changes in endocrine function, chronic diseases, disuse, inflammation, and nutritional deficiencies [2]. Sarcopenia causes physical performance impairment and disability as well as increased risk of falls and fractures [3]. Similarly, aging is usually accompanied by a progressive decrease in bone mineral density (BMD) and bone mass, which is termed osteoporosis if the BMD *T* score is <2.5 standard deviations of the normal *T* scores of young adults [4]. The presence of both sarcopenia and osteoporosis in elderly women is termed osteosarcopenia [5]. A previous study reported that muscle mass loss caused by sarcopenia can reduce the mechanical loading of gravitational forces as well as BMD [6]. The risk of falls and fractures in individuals with a combination of sarcopenia and osteoporosis is higher than in those with either condition alone [5].

Sarcopenia is the age-related progressive loss of muscle mass and impairment of physical performance [7,8]. Providing an effective intervention for patients with sarcopenia is crucial. An earlier study reported that resistance training was used as the primary exercise-based intervention to prevent the progression of muscle mass loss and strength impairment in elderly patients [9]. In addition to resistance training, protein intake is necessary for the maintenance of muscle mass. Protein supplementation is generally considered essential to maximize the skeletal muscle response caused by resistance exercise. Despite discrepancies in the results of studies on the benefits of protein supplementation during resistance exercise interventions, Cermak et al. performed a meta-analysis and reported that protein supplementation increased the lean body mass and muscle strength among both younger and older people [10]. Particularly in frail, older people, protein supplementation can increase the muscle mass during prolonged resistance training [11]. In addition to reducing frailty, protein supplementation combined with resistance training also attenuated the negative effects of sarcopenia and aging on body composition and physical function [12].

Branched-chain amino acids (BCAAs) are obtained from isolated soy protein (ISP) and account for approximately 35% of essential amino acids, which are needed for skeletal muscle formation [13]. BCAAs can shift the net balance of protein metabolism from catabolism to anabolism; therefore, the result is increased protein formation instead of waste [14]. Moreover, BCAAs consisting of the amino acids leucine, isoleucine, and valine have been demonstrated to increase protein anabolism levels and synthesis of skeletal muscle protein; therefore, BCAAs are consumed as nutritional supplements for improving sports performance and preventing the loss of muscle mass caused by aging and illness [15,16,17]. However, another study mentioned the ineffectiveness of BCAA-enriched diet for anabolic effects and that they may be caused by altered distribution and availability of various amino acids resulting from excessive consumption of one or more of the BCAAs [18,19]. Besides, some of the positive effects of BCAA on protein balance are mediated by branched-chain keto acids, glutamine, and beta-hydroxy-beta-methylbutyrate [20]. As a comparison to soy protein, diets supplemented with other types of protein have been studied. In one study, a casein-enriched diet was shown to have no positive effects on protein balance [21]. The anabolic effects of BCAA supplements increase muscle protein synthesis and reduce the degradation of muscle protein. We hypothesized that ISP, which contains BCAAs, combined with resistance exercise training (ET) retards the progress of physical disability and improves muscle mass and bone strength in older postmenopausal women with osteosarcopenia and obesity. Therefore, we conducted this study to investigate the synergistic effects of ISP supplementation and resistance exercise training (ET) on biochemical profiles, exercise performance, and body composition of ovariectomized (OVX) mice with osteosarcopenia and obesity.

## 2. Materials and Methods

### 2.1. Materials, Animals, and Experimental Design

A BCAA-rich ISP supplement in this study was purchased from Best Jet/Gogodone Co. Ltd. (New Taipei City, Taiwan). The nutrients and amino acid categories present in the ISP product were analyzed by SGS Taiwan, Ltd. (New Taipei City, Taiwan) (Table 1). Forty 14-week-old female Institute of Cancer Research (ICR) strain mice raised in specific pathogen-free conditions were purchased (A Charles River Licensee Corp., Yi-Lan, Taiwan). Before the study started, an environment and diet acclimation program was implemented for 1 week. A standard laboratory diet (No. 5001; PMI Nutrition International, Brentwood, MO, USA) and distilled water were provided ad libitum. The mice were entrained to a 12 h light/12 h dark cycle at room temperature (24 ± 1 °C) and 50–60% humidity in this study. The Institutional Animal Care and Use Committee (IACUC) of National Taiwan Sport University reviewed the animal study protocols, and the ethics committee of IACUC approved this study (protocol number 10602). The mice were randomly distributed into five groups (*n* = 8), namely sham OVX (control), OVX, OVX with BCAA-rich ISP supplement (OVX+ISP), OVX with progressive ET (OVX+ET), and OVX with BCAA-rich ISP supplement and ET (OVX+ISP+ET). The OVX procedure was performed within 1 day of purchase by an experienced veterinarian on the mice at 14 weeks of age.

### 2.2. ISP Supplementation

The OVX+ISP+ET group was given ISP 30 min after ET, whereas the ISP group received only the ISP supplement. The recommended dose of ISP for humans is approximately 18.5 g per intake with a normal diet and exercise program. The murine ISP dose (3.8 g/kg) used in this study was determined using a human equivalent dose, which was calculated using body surface area and the following formula from the US Food and Drug Administration:

Assuming a human weight of 60 kg, the human equivalent dose of 18.5 g/60 kg (0.308 g/kg) = 0.308 g.

Next, the conversion coefficient 12.3 was used, and the murine dose was 3.8 g/kg; 12.3 was used to account for differences in body surface area between mice and human beings.

### 2.3. Resistance Exercise Training (ET) Protocol

The OVX mice in the ET and ET+ISP groups were subjected to a standardized protocol, which was modified from previous studies [22,23,24]. The mice were placed in a plastic container (height: 65 cm; diameter: 20 cm) filled with tap water (at 30 ± 1 °C) up to a height of 14–18 cm. The training program consisted of three parts, namely the adaptation, muscle growth, and maximum muscle strength phases. At the beginning of the first week, in the adaptation phase, we subjected the mice to 3 min of rest and 2 min of forced swimming (at a 14-cm depth) with 3–6% body weight (BW) loading for six cycles. The muscle growth phase was observed in the second and third weeks. In this phase, we subjected the OVX mice to 1 min of rest and 1 min of forced swimming (at a 14-cm depth) with 10% to 14% BW loading for five to seven cycles. In the third week, we subjected the mice to 1 min of rest and 1 min of forced swimming (at a 16-cm depth) with 14–17% BW loading for five cycles. In the maximum muscle strength phase, we subjected the mice to 3 min of rest and 0.5 min of forced swimming (at an 18-cm depth) with 22% BW loading for 10 cycles. This training protocol was conducted five times each week.

### 2.4. Forelimb Grip Strength Test

We adopted a low-force testing system (Model-RX-5, Aikoh Engineering, Nagoya, Japan) to evaluate the forelimb grip strength of all the mice in this study. The tensile force data of the mice were recorded using a force transducer, which was equipped with a metal bar (diameter: 2 mm, length: 7.5 cm). Tension equivalent to 10 times the grip strength was the peak tension during each trial, and the tension was recorded using an attached force gauge. The maximal force (grams) of this low-force system was recorded and used to indicate grip strength. The detailed procedures of evaluation have been provided in our previous studies [25,26]. The forelimb grip strength test was performed after administering the ET intervention for 4 weeks.

### 2.5. Swimming Exercise Performance Test

After 4 weeks of intervention, we conducted a swimming exercise performance test to assess the exercise endurance of the mice in this study. A lead sheet (weighing as much as 5% of the average BW of a mouse) was attached to the tail of mice in this exhaustive swimming challenge test. The test was performed in a plastic container (height: 65 cm; radius: 20 cm). The depth of the water was 40 cm, and the temperature was maintained at 27 ± 1 °C. When a mouse failed to rise to the water surface for breathing for >7 s, we considered the mouse to be exhausted, and the duration of swimming was recorded as the exercise endurance.

### 2.6. Clinical Biochemical Profiles

At the end of the experimental period, all the mice were euthanized using 95% CO_2_, and blood was immediately collected during the rest status. Serum was obtained by centrifuging the blood samples, and clinical biochemical variables, such as the levels of aspartate transaminase (AST), alanine transaminase (ALT), alkaline-P, albumin, total protein, blood urea nitrogen (BUN), creatinine, creatine phosphate kinase (CPK), uric acid (UA), total cholesterol (TC), triglycerides (TG), and glucose, were measured using an autoanalyzer (Hitachi 7060, Hitachi, Tokyo, Japan).

### 2.7. Tissue Glycogen and Weight Determination

About 1 h after the last treatment, mice were sacrificed by CO_2_ inhalation. The important visceral organs, including liver, kidney, heart, lung, muscle mass (gastrocnemius and soleus muscles in the posterior part of the lower legs), OPF (ovarian fat pad), and BAT (brown adipocyte tissue) were accurately excised and weighed after sacrifice. Part of the muscle samples were kept in liquid nitrogen for glycogen content analysis. Because the liver and skeletal muscles are two major glycogen storage tissues, we selected these two tissues for glycogen content analysis through the method mentioned in our previous study [26]. At the end of the study, the weights of the related visceral organs and muscles were also recorded for body composition analysis.

### 2.8. Measurement of Bone Strength

The bone strength, stiffness, and energy were assessed in terms of failure load (FL). The FL of the midshaft regions of the right femurs were assessed using a three-point bending test to determine failure (in N) using a computerized testing machine (SV-H1000, Japan Instrumentation System Co., Tokyo, Japan) [27].

### 2.9. Statistical Analysis

Data are presented as means and standard deviations of the means (SD), and one-way analysis of variance was applied to analyze the differences between groups. Statistical analysis was performed using SAS 9.0 (SAS Inst., Cary, NC, USA), and values of *p* < 0.05 indicated statistical significance.

## 3. Results

### 3.1. Effects of BCAA-rich ISP Supplementation and ET on BW and Organ Weights

**Figure 1:**
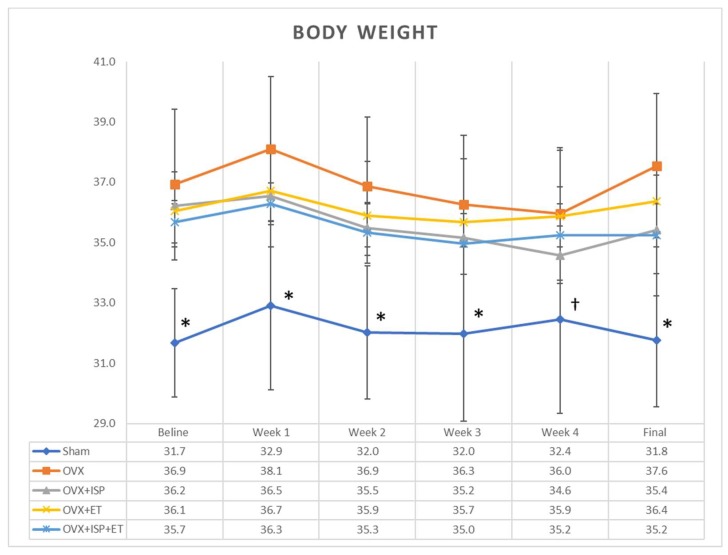
Changes in mean bodyweight (g) during the experiment (*n* = 8). * *p* < 0.05 for sham group compared with OVX, OVX+ISP, OVX+ET, and OVX+ISP+ET groups; † *p* < 0.05 for sham group when compared with OVX and OVX+ET groups by one-way ANOVA. OVX: ovariectomy; ISP: isolated soy protein; ET: exercise training.

All the OVX mice had significantly higher BW than the sham OVX (control) mice throughout the study. The BW changes in all the groups in this study are presented in Figure 1. A comparison of the differences in BWs between OVX groups showed that the initial and final BWs in the OVX, OVX+ISP, OVX+ET, and OVX+ISP+ET groups did not differ significantly. The BW, food consumption, and body composition are presented in Table 2. All the OVX groups exhibited a lower intake of food and water than the sham OVX group.

### 3.2. Effects of ISP Supplementation and ET on Performance on the Forelimb Grip Strength and Exhaustive Swimming Tests

As shown in Table 3, the mean values of forelimb grip strength in the sham OVX, OVX, OVX+ISP, OVX+ET, and OVX+ISP+ET groups were 133, 136, 135, 140, and 143 g, respectively; these groups did not differ significantly in the forelimb grip strength. When the grip strength was calibrated using BWs, no difference was observed among the groups. Furthermore, the exercise endurance did not differ significantly among the sham OVX, OVX, OVX+ISP, OVX+ET, and OVX+ISP+ET groups (Table 3).

### 3.3. Effects of ISP Supplementation and ET on Fatigue-Related Indicators after Acute Exercise

Lactic acid, ammonia, glucose, BUN, and CPK levels can indicate fatigue status after exercise. Lactic acid levels were the lowest and highest in the OVX+ISP+ET and sham OVX groups, respectively. Serum ammonia is usually considered a metabolic product; the serum ammonia levels were lower in the OVX+ISP+ET group than in the other groups. The sham OVX group exhibited higher glucose levels than the other experimental groups. The BUN levels were lower in the sham OVX and OVX groups than in the other experimental groups (*p* < 0.0001). The OVX group exhibited higher CPK levels than the sham OVX (*p* = 0.0291), OVX+ISP (*p* = 0.0347), OVX+ET (*p* = 0.0378), and OVX+ISP+ET (*p* = 0.0062) groups (Table 4).

### 3.4. Effect of ISP Supplementation and ET on Hepatic and Muscular Glycogen Levels

During exercise, glycogen is used as an energy source; therefore, glycogen storage in the liver is associated with physical endurance. However, liver glycogen levels in the OVX group did not differ significantly from those in the other experimental groups. Furthermore, the sham OVX and OVX+ISP groups exhibited higher levels of muscle glycogen than the other experimental groups (Table 4).

### 3.5. Biochemical Analyses at the End of the Experiment

To investigate effects of experimental intervention, we evaluated the biochemical markers at the end of the study. The levels of TG, glucose, UA, and alkaline-P did not differ significantly among the groups in this study (Table 5). The OVX group exhibited higher ALT, AST, creatinine, and CPK levels than the other groups (Table 3). The TC level was higher in the OVX group than in the sham OVX (*p* < 0.0001), OVX+ISP (*p* = 0.0124), and OVX+ISP+ET (*p* = 0.0129) groups but was similar to that in the OVX+ET group (*p* = 0.0536). The OVX+ISP and OVX+ISP+ET groups exhibited higher BUN levels than the sham OVX, OVX, and OVX+ET groups. The OVX group exhibited higher lactate dehydrogenase (LDH) levels than the sham OVX (*p* = 0.0256), OVX+ISP (*p* = 0.0173), and OVX+ISP+ET (*p* = 0.0089) groups. The high-density lipoprotein (HDL-c) levels in the OVX+ISP, OVX+ET, and OVX+ISP+ET groups were higher than those in the sham OVX and OVX groups. The low-density lipoprotein (LDL-c) levels in the sham OVX group were higher than those in the OVX (*p* = 0.0399), OVX+ISP (*p* = 0.0013), OVX+ET (*p* = 0.0246), and OVX+ISP+ET (*p* = 0.0283) groups (Table 5).

### 3.6. Bone Strength at the End of the Experiment

Our study investigated bone strength and stiffness at the end of the experiment. The bone energy values of the sham OVX, OVX+ISP, OVX+ET, and OVX+ISP+ET groups did not differ significantly. However, the bone energy value in the OVX group was lower than those in the sham OVX (*p* = 0.0299) and OVX+ISP+ET (*p* = 0.0299) groups. The sham OVX group exhibited higher levels of bone stiffness than the OVX (*p* = 0.0116), OVX+ISP (*p* = 0.0183), OVX+ET (*p* = 0.0071), and OVX+ISP+ET (*p* = 0.0433) groups. Furthermore, no statistical difference was observed among any of the OVX groups with or without intervention in this study. The OVX+ISP+ET and sham OVX groups exhibited higher bone strength than the OVX, OVX+ISP, and OVX+ET groups (Table 6).

## 4. Discussion

In this study, we found that four weeks of ISP supplementation and ET increased the bone strength in the OVX mice. However, the improvements in the muscle mass, forelimb grip strength in the intervention groups were not significantly higher than those in the OVX group. The laboratory data (of fatigue-related biomarkers) of the OVX+ISP+ET group indicated lower fatigue levels than those in the other groups. The OVX+ISP+ET group did not exhibit higher endurance than the other groups in the swimming tests, but the fatigue biomarkers indicated lower levels of fatigue in the OVX+ISP+ET group than in the other groups.

Our study demonstrated that OVX increased BW and body fat accumulation in the experimental groups. Estrogen deficiency caused by OVX increased the food intake of the mice. Estradiol controls the food intake through feedback signal regulation; therefore, it affects BW and the amount of food consumed [28]. This finding is compatible with our study, which presented significantly higher weight gain in the OVX groups than in the sham OVX group.

The OVX group did not show an increase in the amount of food consumed; however, the mice in the OVX group showed an increase in BW. The increase in weight could be related to menopausal transition in the OVX mice; a previous study had mentioned that menopausal transition is accompanied by a decrease in food intake [29]. The OVX mice exhibited greater muscle mass than the other groups; however, the amount of brown adipose tissue did not differ among the groups. The OVX+ISP and OVX+ET groups exhibited less muscle mass than the OVX group. The difference in muscle mass can be explained using the relationship between BW load and the maintenance of muscle mass; moreover, a previous study had mentioned that weight loss accelerates age-related muscle decline [30]. Contrary to our expectation, the effects of exercise and BCAA-rich ISP supplement on muscle mass (increasing muscle mass) were not significant in this four-week intervention. Findings from previous studies have shown that both resistance training and protein supplementation are less effective in older adults than in younger adults; this is known as chronic blunting responsiveness in older people [31,32,33]. Furthermore, a recent study indicated that supplementation with protein or essential amino acids did not augment the effect of progressive resistance ET on body composition, muscle strength, size, or functional ability among older adults [34]. The blunting response could have caused the lack of difference in muscle mass and body composition among all the OVX groups with or without ISP supplementation or ET intervention.

The forelimb grip strength measures maximal and explosive force production, and the swimming test evaluates the aerobic capacity of the mice. In our study, grip strength and swimming endurance did not improve in any of the OVX groups. We considered that the aforementioned blunting response in the older mice could have caused a low-level response to ISP supplementation and ET intervention for four weeks. We supposed that the intensity of exercise and amount of nutritional supplement provided could not improve the physical performance of the OVX mice.

The status of muscle fatigue after exercise can be assessed using levels of lactate, ammonia, glucose, CPK, and BUN [35]. Lactate is an oxidized substrate in the skeletal muscles, a precursor for gluconeogenesis in the muscles, and is produced through glycolysis. Lactic acid is formed from lactate; high levels of lactic acid after energy utilization indicate poor exercise endurance. Our study showed that the OVX+ISP or OVX+ET groups accumulated lower levels of lactic acid than the OVX and sham OVX groups. Moreover, because of the synergistic effects of the ISP supplements with ET, the OVX+ISP+ET group exhibited the lowest lactic acid levels. However, the swimming endurance results of the intervention groups were not superior to those of the OVX group. This finding can support our hypothesis that the amount of ISP supplement used or the exercises selected were inadequate for improvement in physical performance. A previous study had mentioned that peripheral and central fatigue levels are related to increased ammonia levels during exercise [36]. The OVX+ISP+ET group exhibited insignificantly lower levels of ammonia than the OVX and OVX+ISP groups. We supposed that ET reduced the ammonia level in the OVX+ISP+ET group. CPK levels indicate muscle injuries, which are caused by muscular dystrophy, severe muscle breakdown, myocardial infarction, autoimmune myositis, and acute renal failure. Our study showed that the OVX+ISP, OVX+ET, and OVX+ISP+ET groups exhibited lower CPK levels than the OVX group. Both ISP supplementation and ET can improve the laboratory data of CPK. However, the combination of ISP supplementation and ET did not show an additive effect on the CPK levels in the OVX group.

Aging aggravates bone loss in menopausal women because of the loss of estrogen. We simulated this condition through bilateral OVX. The combination of muscle loss and osteoporosis is called osteosarcopenia. Our study demonstrated that bone strength was higher in the OVX+ISP+ET group than in the OVX group and similar to that in the sham OVX group. However, the bone stiffness did not differ among the groups. ET is considered as a method for increasing bone mass using stress induced by mechanical loading, inhibiting bone resorption, and increasing bone formation [37]. However, contrary to our expectations, the ET intervention did not significantly improve bone strength or stiffness in the OVX mice. The effect of ET on bone strength in the OVX mice might have been attenuated because the intensity and progression dosage were insufficient to change bone stiffness. Our results revealed an additive effect between ISP supplementation and ET intervention on bone strength. Thus far, evidence indicating that ISP supplementation influences bone strength and structure is not available. The mechanism of maintaining bone strength in OVX-induced menopausal osteoporosis in mice requires detailed investigation; consequently, additional studies are needed in the future.

## 5. Conclusions

In conclusion, our study demonstrated that BCAA-rich ISP supplementation and ET improved bone strength in OVX mice. Although the grip strength and exercise endurance performance of the treated mice did not improve significantly, the levels of exercise-induced fatigue-related biomarkers, such as lactic acid, ammonia, and CPK, improved with concurrent ISP+ET intervention in OVX mice. The lipid profile results of the OVX+ISP and OVX+ET groups were superior to those of the OVX group. OVX caused an increase in BW of the mice, but the body composition of muscle mass was not higher in the OVX+ISP, OVX+ET, or OVX+ISP+ET groups than in the OVX group. Although improvements in strength and endurance after ET and ISP supplementation were not observed in this study, we found that the combination of interventions reduced the levels of fatigue-related biomarkers in the OVX mice. Our study revealed the potential additive effects of BCAA-rich ISP and ET on improving bone strength and levels of fatigue-related parameters in OVX mice. Additional studies on ISP supplementation and ET for older postmenopausal women are recommended in the future.

## Figures and Tables

**Table 1:** Nutrients, hydrolyzed amino acid profiles, total branched-chain amino acids (BCAAs), and isoflavones of ISP.

Nutrients	Content/40 g ISP
Protein	23.2 g
Fat	0.8 g
Saturated fat	0.2 g
Trans fat	0 g
Carbohydrate	11 g
Sugar	10.6 g
Dietary fiber	0.4 g
Sodium	174 mg
Total calories	144.2 Kcal
**Hydrolyzed amino acid profiles**	g/40 g
Leucine	1.08
Valine	1.14
Isoleucine	1.20
Cystine	0.26
Tryptophan	0.27
Methionine	0.28
Threonine	0.49
Histidine	0.63
Tyrosine	0.79
Alanine	0.88
Glycine	0.94
Serine	1.12
Proline	1.18
Phenylalanine	1.26
Lysine	1.50
Arginine	1.89
Aspartic acid	2.69
Glutamic acid	4.81
**Isoflavones**	mg/40 g
Daidzin	1.5
Daidzein	2.0
Genistin	4.5
Genistein	4.0

**Table 2:** Effect of ISP supplementation and four weeks of ET on body composition at the end of the experiment.

Characteristic	Sham OVX	OVX	OVX+ISP	OVX+ET	OVX+ISP+ET
Initial BW (g)	31.7 ± 1.89 ^a^	36.9 ± 5.10 ^b^	36.2 ± 2.36 ^b^	36.1 ± 1.33 ^b^	35.7 ± 0.66 ^b^
Final BW (g)	31.8 ± 2.18 ^a^	37.6 ± 4.89 ^b^	35.4 ± 2.12 ^b^	36.4 ± 2.22 ^b^	35.2 ± 1.99 ^b^
Food intake (g/day)	6.31 ± 0.73 ^d^	4.80 ± 0.91 ^bc^	4.35 ± 0.73 ^a^	4.61 ± 0.69 ^ab^	5.09 ± 0.75 ^c^
Water intake (mL/day)	7.43 ± 1.02 ^d^	6.50 ± 0.94 ^a^	5.40 ± 0.64 ^b^	5.95 ± 0.64 ^c^	6.19 ± 0.83 ^ac^
Weight (g)					
Liver	1.52 ± 0.21 ^a^	1.78 ± 0.40 ^b^	1.66 ± 0.15 ^ab^	1.64 ± 0.15 ^ab^	1.79 ± 0.03 ^b^
Kidney	0.45 ± 0.09 ^a^	0.45 ± 0.07 ^a^	0.42 ± 0.03 ^a^	0.42 ± 0.03 ^a^	0.44 ± 0.03 ^a^
OFP	0.27 ± 0.12 ^a^	0.61 ± 0.21 ^b^	0.65 ± 0.28 ^b^	0.64 ± 0.31 ^b^	0.50 ± 0.28 ^ab^
Heart	0.20 ± 0.04 ^a^	0.21 ± 0.04 ^a^	0.18 ± 0.02 ^a^	0.18 ± 0.03 ^a^	0.18 ± 0.04 ^a^
Lung	0.26 ± 0.04 ^a^	0.25 ± 0.05 ^ab^	0.20 ± 0.02 ^b^	0.22 ± 0.02 ^b^	0.22 ± 0.02 ^b^
Muscle	0.32 ± 0.02 ^a^	0.38 ± 0.05 ^c^	0.34 ± 0.04 ^ab^	0.34 ± 0.03 ^ab^	0.35 ± 0.03 ^bc^
BAT	0.12 ± 0.02 ^a^	0.14 ± 0.03 ^ab^	0.12 ± 0.01 ^a^	0.15 ± 0.04 ^a^	0.13 ± 0.03 ^ab^
Intestine	3.00 ± 0.80 ^a^	3.80 ± 0.21 ^b^	3.34 ± 0.21 ^ab^	3.32 ± 0.29 ^ab^	3.60 ± 0.40 ^b^
Relative weight (%)					
Liver	4.8 ± 0.53 ^a^	4.7 ± 0.69 ^a^	4.7 ± 0.35 ^a^	4.5 ± 0.30 ^a^	4.9 ± 0.08 ^a^
Kidney	1.4 ± 0.25 ^b^	1.2 ± 0.10 ^a^	1.2 ± 0.10 ^a^	1.2 ± 0.06 ^a^	1.2 ± 0.05 ^a^
OFP	0.8 ± 0.36 ^a^	1.6 ± 0.53 ^b^	1.8 ± 0.71 ^b^	1.7 ± 0.79 ^b^	1.4 ± 0.84 ^ab^
Heart	0.6 ± 0.15 ^b^	0.6 ± 0.10 ^ab^	0.5 ± 0.05 ^a^	0.5 ± 0.09 ^a^	0.5 ± 0.11 ^a^
Lung	0.8 ± 0.14 ^b^	0.7 ± 0.12 ^a^	0.6 ± 0.04 ^a^	0.6 ± 0.04 ^a^	0.6 ± 0.05 ^a^
Muscle	0.3 ± 0.02 ^a^	0.4 ± 0.05 ^c^	0.3 ± 0.04 ^ab^	0.3 ± 0.03 ^ab^	0.4 ± 0.03 ^bc^
BAT	0.4 ± 0.05 ^a^	0.4 ± 0.06 ^a^	0.3 ± 0.05 ^a^	0.4 ± 0.09 ^b^	0.3 ± 0.06 ^a^
Intestine	9.5 ± 1.19 ^a^	10.0 ± 0.73 ^a^	9.5 ± 0.73 ^a^	9.1 ± 0.81 ^a^	9.9 ± 1.03 ^a^

Data are mean ± SD for *n* = 8 mice in each group. Values in the same line with different superscripts letters (a, b, c, d) differ significantly, *p* < 0.05 through one-way analysis of variance. Muscle mass includes both the gastrocnemius and soleus muscles in the posterior part of the lower legs. BW: body weight; BAT: brown adipose tissue; OFP: ovarian fat pad; OVX: ovariectomized; ET: exercise training; ISP: isolated soy protein supplementation; SD: standard deviation.

**Table 3:** Effect of ISP and ET on forelimb grip strength and exhaustive swimming test.

Characteristic	Sham OVX	OVX	OVX+ISP	OVX+ET	OVX+ISP+ET
Grip strength (g)	133 ± 21	136 ± 44	135 ± 17	140 ± 20	143 ± 18
Grip strength (%)	417 ± 69	388 ± 44	400 ± 55	403 ± 61	427 ± 52
Swimming time (min)	39.9 ± 48.0	29.4 ± 29.7	53.4 ± 39	48.5 ± 20.7	52.0 ± 29.2

Data are mean ± SD, (*n* = 8). No significant differences at *p* < 0.05 through one-way analysis of variance. OVX: ovariectomized; ET: exercise training; ISP: isolated soy protein supplementation; SD: standard deviation.

**Table 4:** Effect of ISP supplementation and ET on fatigue-related indicators and glycogen level after acute exercise.

Characteristic	Sham OVX	OVX	OVX+ISP	OVX+ET	OVX+ISP+ET
Ammonia (umol/L)	67 ± 11 ^b^	84 ± 13 ^c^	89 ± 14 ^c^	59 ± 7 ^ab^	52 ± 4 ^a^
GLU (mg/dL)	163 ± 26 ^c^	125 ± 16 ^b^	117 ± 15 ^ab^	139 ± 20 ^b^	102 ± 10 ^ab^
LACT (mmol/L)	9.1 ± 0.38 ^d^	8.0 ± 1.25 ^c^	6.4 ± 1.08 ^b^	6.3 ± 0.85 ^b^	4.2 ± 0.27 ^a^
BUN (mg/dL)	17.7 ± 3.1 ^a^	18.9 ± 2.5 ^a^	41.6 ± 1.7 ^c^	31.5 ± 2.4 ^b^	39.2 ± 4.6 ^c^
CPK (U/L)	376 ± 161 ^a^	643 ± 419 ^b^	386 ± 201 ^a^	390 ± 109 ^a^	284 ± 96 ^a^
GLY (mg/g liver)	40.0 ± 11.66 ^b^	33.5 ± 8.50 ^ab^	24.1 ± 9.35 ^a^	27.3 ± 7.12 ^a^	33.5 ± 15.94 ^ab^
GLY (mg/g muscle)	1.2 ± 0.22 ^b^	1.0 ± 0.22 ^a^	1.2 ± 0.17 ^b^	0.9 ± 0.16 ^a^	0. 9± 0.12 ^a^

Data are mean ± SD, (*n* = 8). Different letters (a, b, c, d) in the same row indicate a significant difference at *p* < 0.05 through one-way analysis of variance. GLU: glucose; LACT: lactate; BUN: blood urine nitrogen; CPK: creatine phosphate kinase; GLY: glycogen; OVX: ovariectomized; ET: exercise training; ISP: isolated soy protein supplementation; SD: standard deviation.

**Table 5:** Effect of ISP supplementation and four weeks of ET on biochemical serum levels at the end of the experiment.

Characteristic	Sham OVX	OVX	OVX+ISP	OVX+ET	OVX+ISP+ET
AST (U/L)	96 ± 14 ^a^	119 ± 27 ^b^	98 ± 11 ^a^	98 ± 11 ^a^	96 ± 15 ^a^
ALT (U/L)	51 ± 6 ^a^	70 ± 25 ^b^	54 ± 9 ^a^	56 ± 12 ^a^	51 ± 9 ^a^
Alkaline-P	62 ± 11 ^a^	57 ± 10 ^a^	62 ± 12 ^a^	60 ± 7 ^a^	58 ± 11 ^a^
CPK (U/L)	335 ± 196 ^ab^	584 ± 319 ^c^	325 ± 162 ^ab^	497 ± 171 ^bc^	280 ± 133 ^a^
Albumin (g/dL)	3.1 ± 0.1 ^a^	3.2 ± 0.1 ^b^	3.2 ± 0.1 ^b^	3.2 ± 0.1 ^b^	3.3 ± 0.1 ^b^
TP (g/dL)	5.0 ± 0.12 ^a^	5.0 ± 0.18 ^a^	5.1 ± 0.26 ^ab^	5.2 ± 0.10 ^b^	5.2 ± 0.05 ^b^
BUN (mg/dL)	13.1 ± 1.39 ^a^	18.9 ± 2.29 ^b^	30.4 ± 6.21 ^c^	20.6 ± 2.15 ^b^	27.1 ± 2.08 ^c^
Creatinine (mg/dL)	0.18 ± 0.04 ^a^	0.28 ± 0.04 ^c^	0.19 ± 0.04 ^a^	0.26 ± 0.03 ^bc^	0.24 ± 0.05 ^b^
UA (mg/dL)	1.08 ± 0.15 ^a^	1.08 ± 0.30 ^a^	1.15 ± 0.25 ^a^	1.05 ± 0.20 ^a^	1.04 ± 0.23 ^a^
Glucose (mg/dL)	121 ± 17 ^a^	121 ± 8 ^a^	115 ± 4 ^a^	121 ± 9 ^a^	122 ± 10 ^a^
TG (mg/dL)	79 ± 12 ^a^	74 ± 20 ^a^	72 ± 12 ^a^	78 ± 7 ^a^	69 ± 17 ^a^
TC (mg/dL)	89 ± 10 ^a^	125 ± 24 ^c^	105 ± 13 ^b^	110 ± 15 ^bc^	105 ± 10 ^b^
HDL-c	63 ± 10 ^a^	71 ± 9 ^a^	88 ± 7 ^b^	83 ± 11 ^b^	90 ± 5 ^b^
LDL-c	9.8 ± 2.4 ^b^	8.0 ± 2.0 ^a^	6.9 ± 1.0 ^a^	7.8 ± 1.2 ^a^	7.9 ± 1.0 ^a^
LDH	343 ± 46 ^a^	420 ± 96 ^b^	337 ± 41 ^a^	390 ± 77 ^ab^	328 ± 55 ^a^

Data are mean ± SD, (*n* = 8). Different letters (a, b, c) in the same row indicate a significant difference at *p* < 0.05 through one-way analysis of variance. AST, aspartate aminotransferase; ALT, alanine aminotranferease; CPK, creatine phosphate kinase; TP, total protein; BUN, blood urea nitrogen; UA, uric acid; TC; total cholesterol; TG, triacylglycerol; HDL-c; high-density lipoprotein; LDL-c, low-density lipoprotein; LDH, lactate dehydrogenase; OVX: ovariectomized; ET: exercise training; ISP: isolated soy protein supplementation; SD: standard deviation.

**Table 6:** Bone strength at the end of the experiment.

Characteristic	Sham OVX	OVX	OVX+ISP	OVX+ET	OVX+ISP+ET
Bone energy (mJ)	5.4 ± 1.25 ^b^	3.9 ± 0.68 ^a^	4.5 ± 0.87 ^ab^	5.1 ± 2.0 ^ab^	5.4 ± 1.71 ^b^
Bone stiffness (N/mm)	113.5 ± 15.37 ^b^	94.4 ± 10.76 ^a^	95.8 ± 13.07 ^a^	93.0 ± 16.12 ^a^	98.5 ± 15.63 ^a^
Bone strength (N)	29.0 ±4.44 ^c^	23.6 ± 3.50 ^a^	25.1 ± 2.85 ^ab^	25.4 ± 1.30 ^ab^	29.0 ± 3.02 ^bc^

Data are mean ± SD, (*n* = 8). Different letters (a, b, c) in the same row indicate a significant difference at *p* < 0.05 through one-way analysis of variance. OVX: ovariectomized; ET: exercise training; ISP: isolated soy protein supplementation; SD: standard deviation; N: newton.
